# Supplementary material for: Deletion of Topoisomerase 1 in excitatory neurons causes genomic instability and early onset neurodegeneration
Source: Nat Commun. 2020 Apr 23;11:1962. doi: 10.1038/s41467-020-15794-9 (PMC7181881; doi:10.1038/s41467-020-15794-9)
Supplement: Supplementary file 1 — Supplementary Information [file 41467_2020_15794_MOESM1_ESM.pdf]

Supplementary Information

Deletion of Topoisomerase 1 in excitatory neurons causes genomic instability and early onset neurodegeneration

Fragola et al.

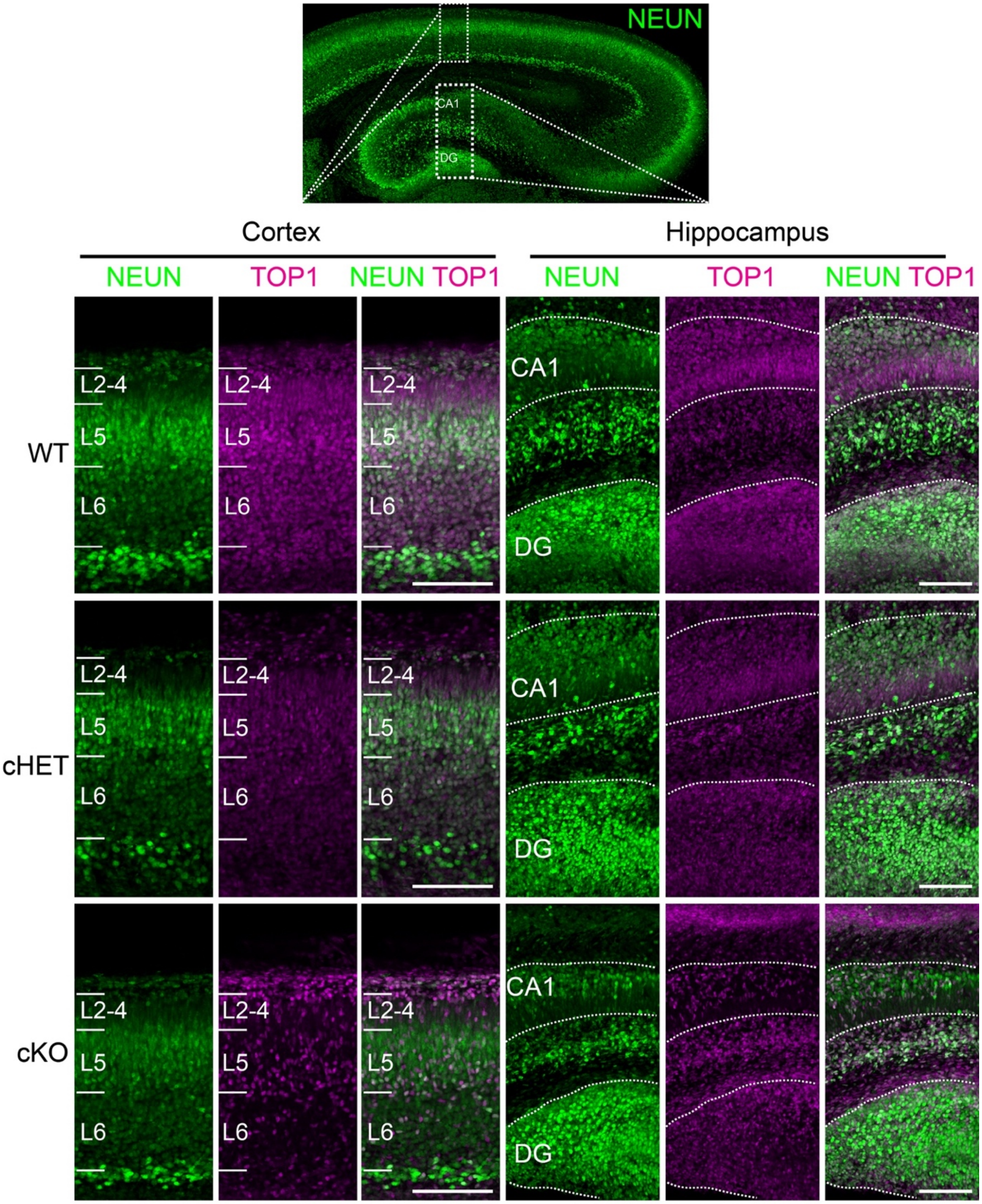

Supplementary Figure 1. TOP1 expression in WT, *Top1* cHET and *Top1* cKO P0 brain. Immunostaining of TOP1 and NEUN in P0 cortex and hippocampus of WT, *Top1* cHET and *Top1* cKO mice. Inset scale bars = 100 µm. DG = dentate gyrus. Images are representative of 2 independent experiments.

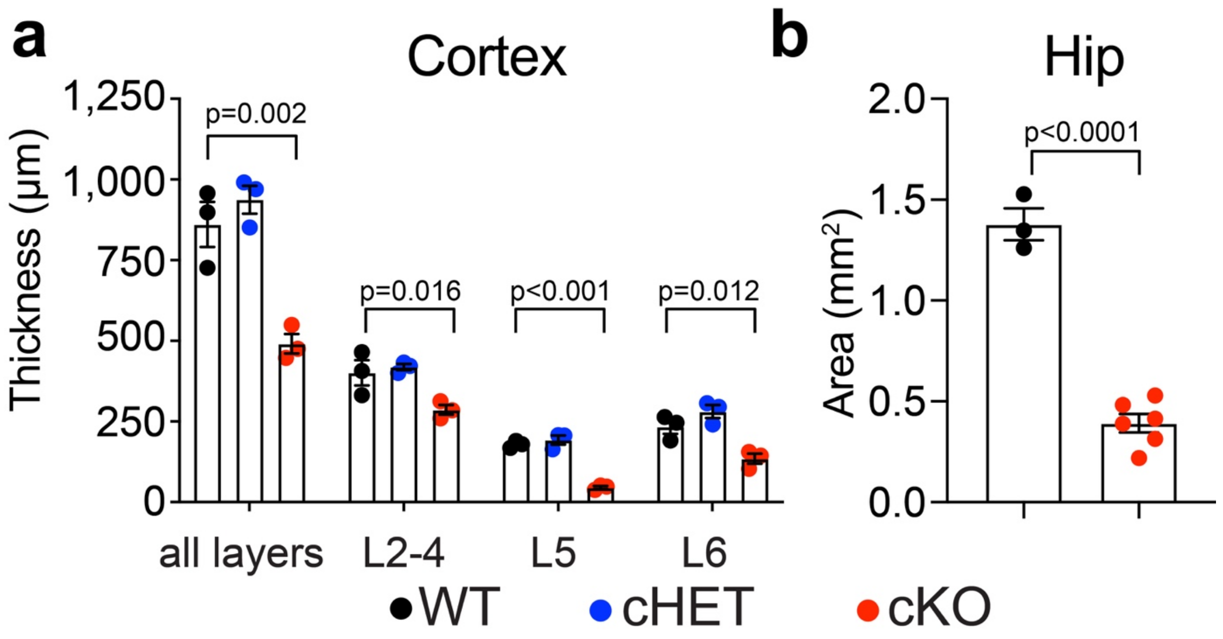

Supplementary Figure 2. Cortical thickness in all layers and hippocampal area are reduced in *Top1* cKO mice at P15.

(a) Quantification of cortical thickness in WT, *Top1* cHET and *Top1* cKO (n=3 mice per genotype). One-way ANOVA. **(b)** Quantification of hippocampal (Hip) area.

WT (n=3), cKO (n=6). Two-sided student t-test. Values are mean and error bars are ± SEM.

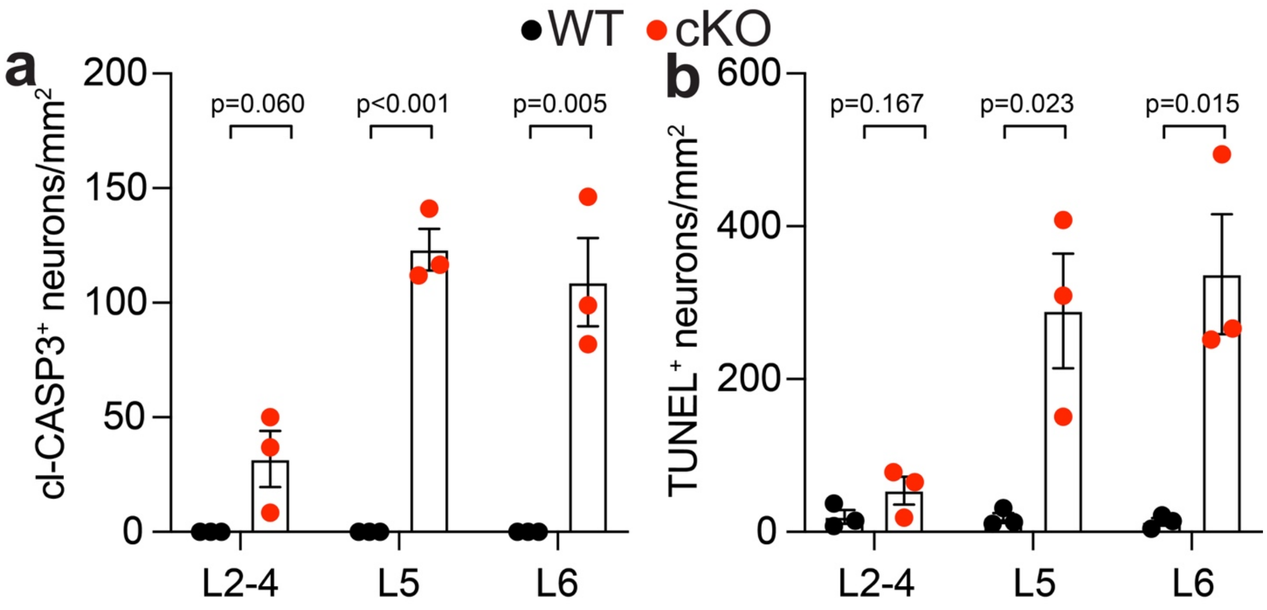

Supplementary Figure 3. Increased apoptosis of L5 and L6 neurons in *Top1* cKO mice.

Quantification of **(a)** cl-CASP3^{+} and **(b)** TUNEL^{+} neurons in cerebral cortex L2-4, L5 and L6 of WT (n=3) and cKO (n=3) P7 mice. n=2 sections per mouse. Two-sided student's t-test. Values are mean and error bars are ± SEM.

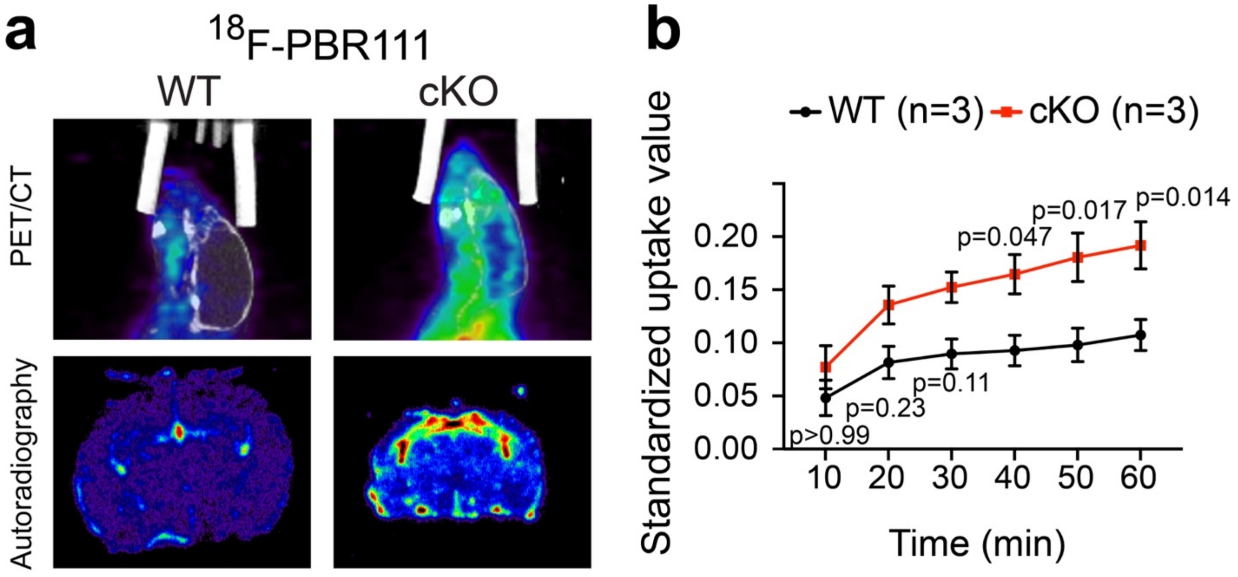

Supplementary Figure 4. Neuroinflammation in *Top1* cKO mice visualized by PET imaging with ^{18}F-PBR111.

(a) PET and computed tomography (CT) images (upper), high resolution autoradiography (lower) of WT and cKO P15 mice injected i.p. with ^{18}F-PBR111. **(b)** Quantification of ^{18}F-PBR111 uptake in the brain of WT and cKO P15 mice. Two-way ANOVA with Bonferroni post hoc test. 95% confidence interval of difference: 10 min (-0.100 to 0.0423), 20 min (-0.125 to 0.0170), 30 min (-0.134 to 0.00840), 40 min (-0.143 to -0.000815), 50 min (-0.154 to -0.0113), 60 min (-0.156 to -0.0132). n=3 mice per genotype. Values are mean and error bars are ± SEM.

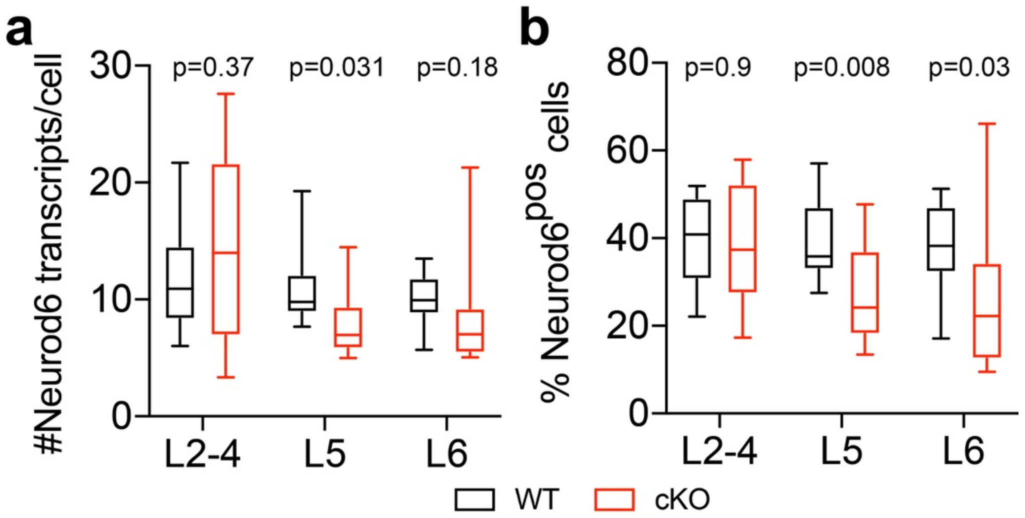

Supplementary Figure 5. Quantification of *Neurod6* transcript levels and percentage of *Neurod6*$^{+}$ neurons in different cortical layers with single-molecule *in situ* hybridization.

(a) Average number of transcripts/cell/section from the indicated cortical layers in P7 WT and *Top1* cKO cortex. **(b)** Mean percentages of neurons with more than 5 transcripts (*Neurod6*$^{+}$) in the different cortical layers of WT and cKO cortex. Box is first to third quartile, line is median, error bars are minimum and maximum values. Two-sided student t-test. n=11 WT sections, n=14 cKO sections. 2 mice/genotype.

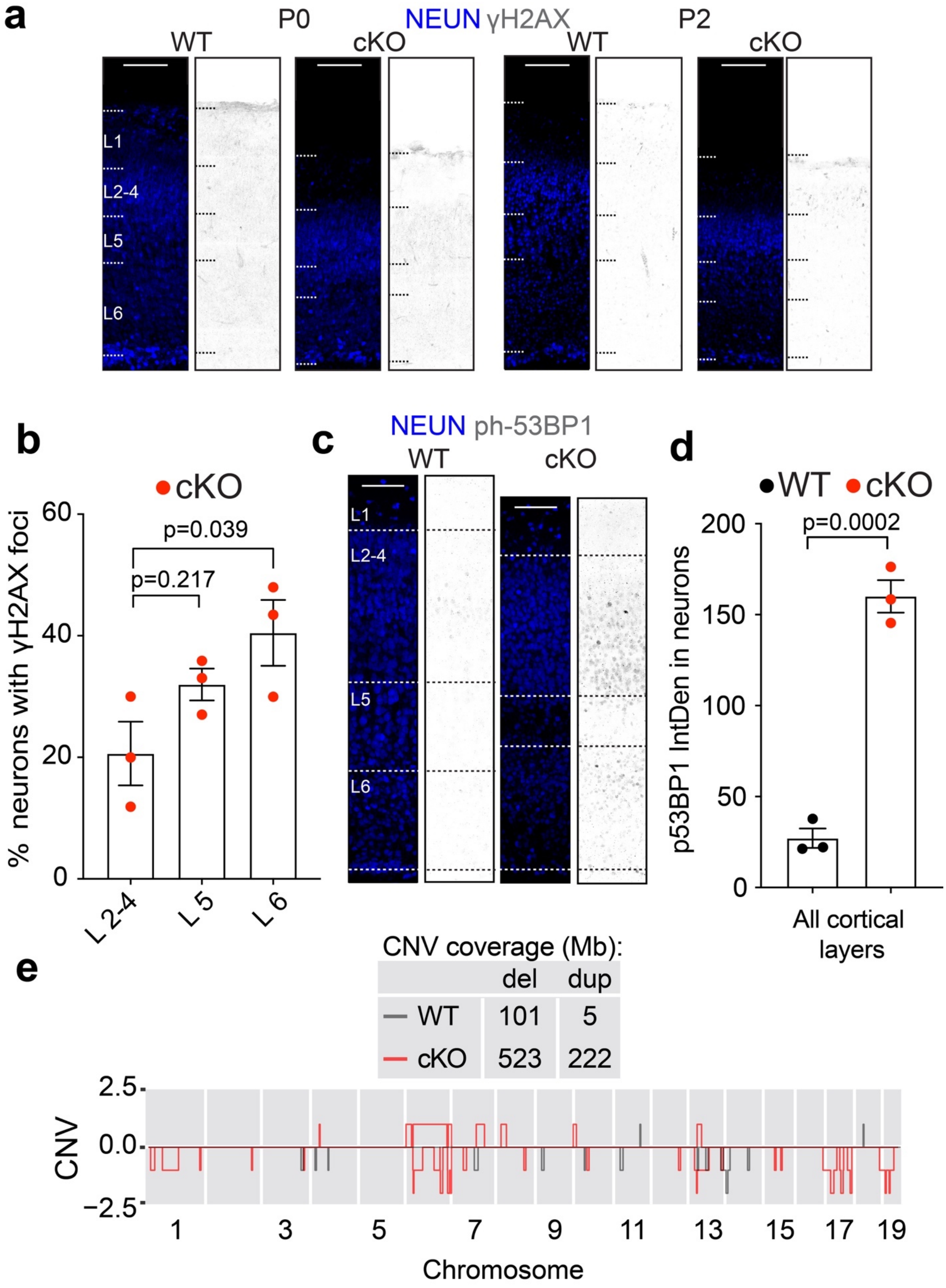
a
P0
NEUN γH2AX
P2
WT
cKO
WT
cKO
L1
L2-4
L5
L6
b
cKO
p=0.039
p=0.217
% neurons with γH2AX foci
60
40
20
0
L 2-4
L 5
L 6
c
NEUN ph-53BP1
WT
cKO
L1
L2-4
L5
L6
d
WT
cKO
p=0.0002
p53BP1 IntDen in neurons
200
150
100
50
0
All cortical layers
e
CNV coverage (Mb):
del dup
WT 101 5
cKO 523 222
CNV
2.5
0.0
−2.5
1
3
5
7
9
11
13
15
17
19
Chromosome

Supplementary Figure 6. Elevated DNA damage and more abundant CNVs in P7 *Top1* cKO mice.

(a) Immunostaining and of γH2AX and NEUN in P0 and P2 WT and cKO cortex. Images are representative of 3 mice per genotype. Scale bar = 100 μm. **(b)** Graph showing the percentage of neurons with γH2AX foci in L2-4, L5 and L6 of *Top1* cKO mice (n=3 mice per genotype). One-way ANOVA with Dunnett's multiple comparison test. **(c)** Immunostaining and **(d)** quantification of ph-53BP1 and NEUN in P7 WT and cKO cortex. (n=3 mice per genotype). IntDen=Integrated density. Scale bar = 100 μm. Two-sided student t-test. **(e)** Graph showing the overlap of all the CNVs identified in WT and cKO neurons at the different genomic locations**.** CNV coverage is indicated. Y axis indicates the number of CNVs identified at the correspondent genomic location; above zero are duplications, below zero are deletions. Values are mean and error bars are ± SEM.

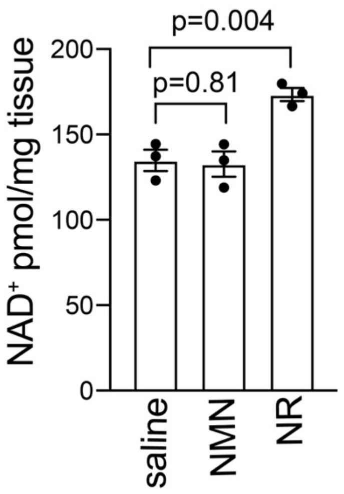

Supplementary Figure 7. Treatment with NR increases NAD⁺ levels in the cerebral cortex of neonatal mice.

Quantification of NAD⁺ levels in cortical lysates of P3 mice collected 1 h after i.p. injection with saline, NR (100 mg/kg) or NMN (100 mg/kg). One-way ANOVA. n=3 animals per condition. Values are mean and error bars are ± SEM.

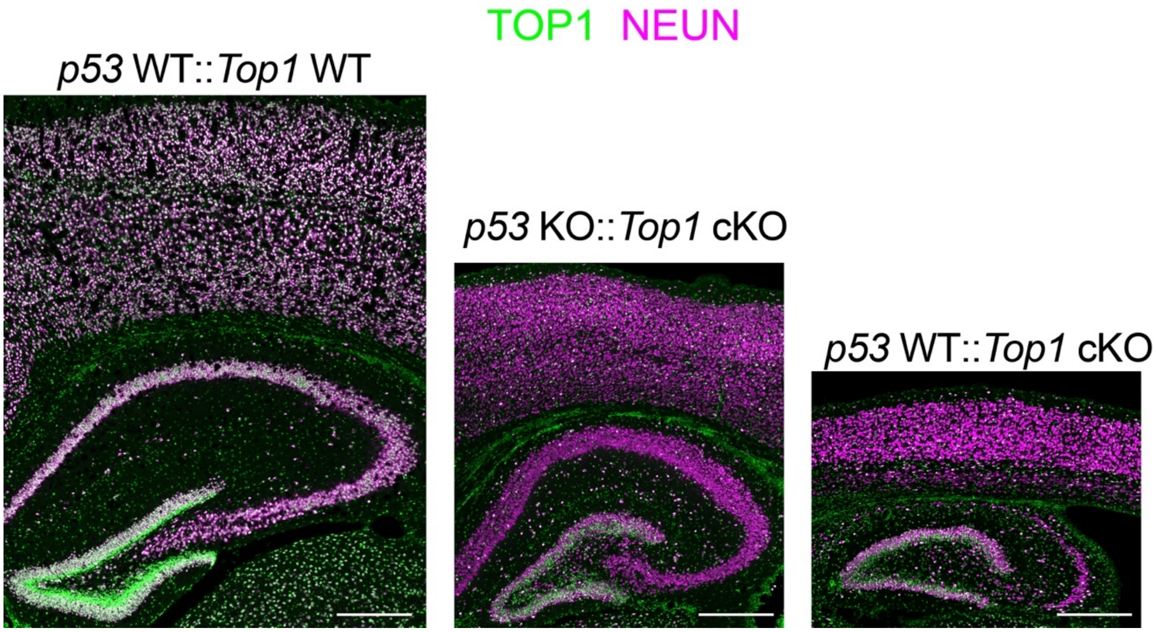

Supplementary Figure 8. Increased neuronal survival in *p53 KO::Top1* cKO P15 cortex and hippocampus.

TOP1 and NEUN immunostaining of *p53 WT::Top1 WT*, *p53 KO::Top1 cKO* and *p53 WT::Top1 cKO* P15 cortex. Scale bar = 300 μm. Images are representative of 3 independent experiments.

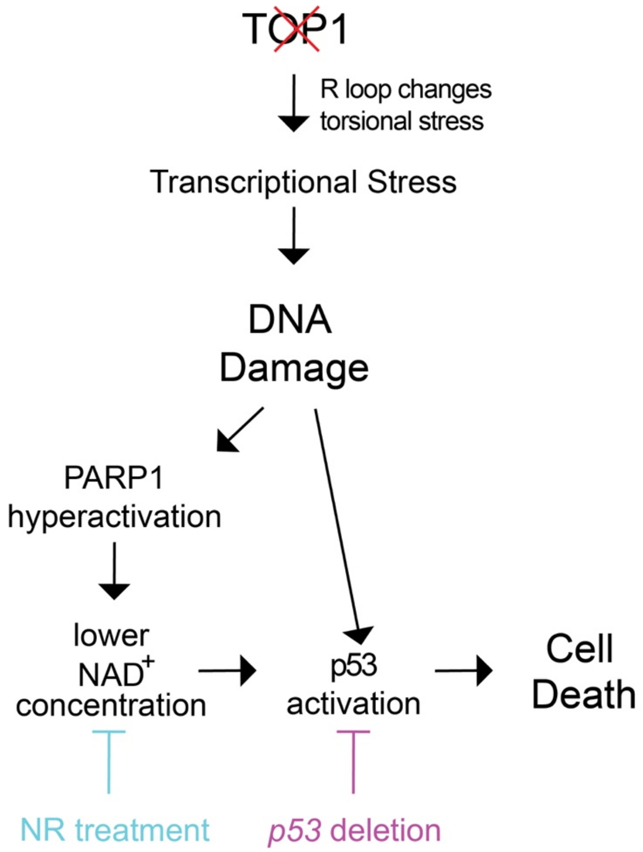

Supplementary Figure 9. Potential mechanism of DNA damage-induced neuronal death in *Top1*-deficient neurons.

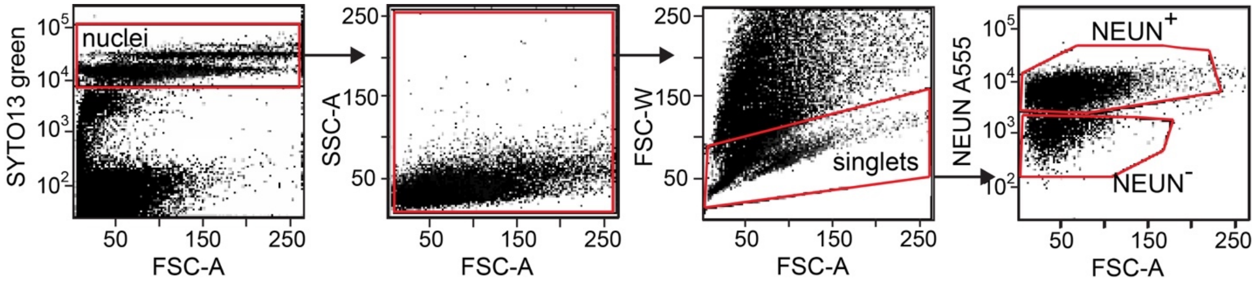

Supplementary Figure 10. Gating strategy used for sorting neurons for Figure 6d and Supplementary Figure 6e.

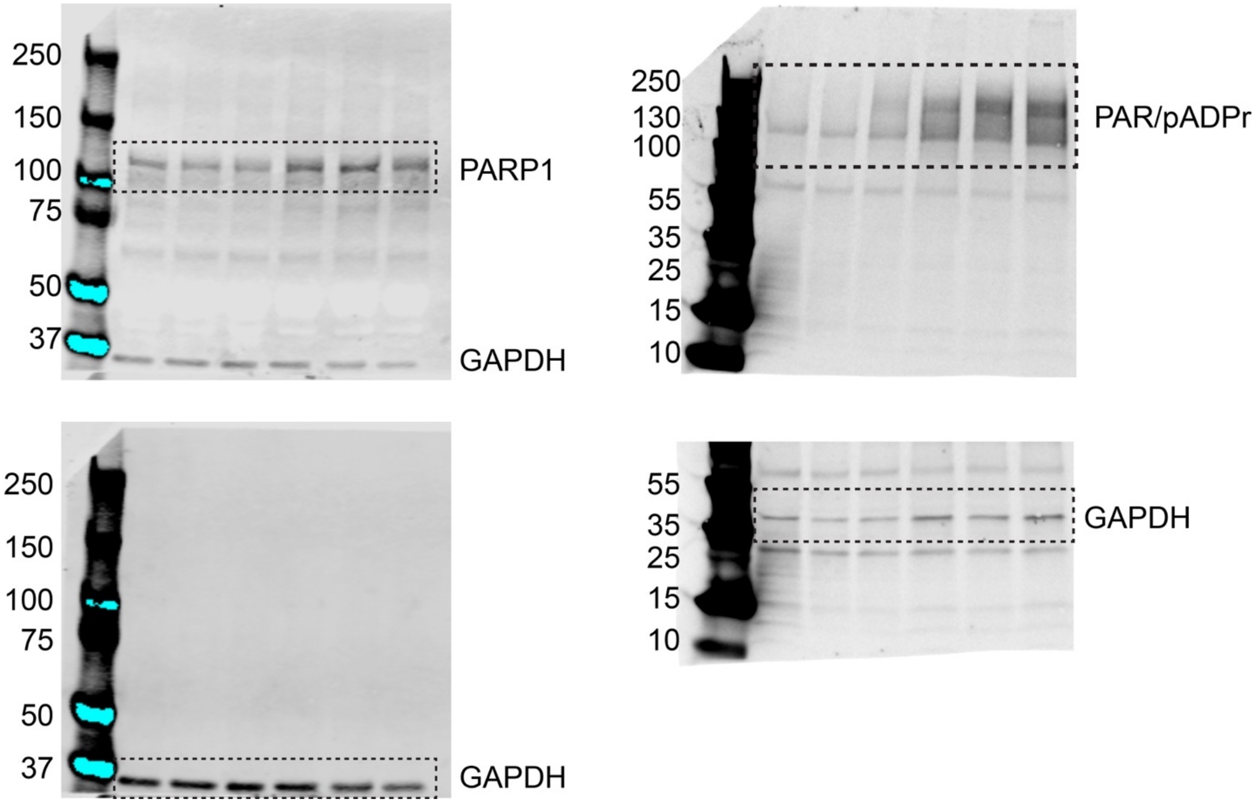

Supplementary Figure 11. Uncropped immunoblots from Figure 7a.
