# Supplementary material for: Deletion of Topoisomerase 1 in excitatory neurons causes genomic instability and early onset neurodegeneration
Source: Nat Commun. 2020 Apr 23;11:1962. doi: 10.1038/s41467-020-15794-9 (PMC7181881; doi:10.1038/s41467-020-15794-9)
Supplement: Supplementary file 3 — Description of Additional Supplementary Files [file 41467_2020_15794_MOESM3_ESM.docx]

**Description of Supplementary Data**

**Supplementary Data 1. Single-cell RNA-seq cluster marker genes.** Cluster 1, 2 and 3 marker genes obtained by a presence-absence binomial test (mean log_2_ fold-change > 1.0, p-value < 0.05). Gene names, log2 fold changes and p-values are indicated.

**Supplementary Data 2. List of differentially expressed genes in the excitatory neuron cluster identified by single-cell RNA-seq.** Gene names, normalized expression, log_2_ fold-changes and adj. p-values calculated by DESeq2 are indicated. Long gene classification (blue if >100 kb) is indicated.

**Supplementary Data 3. Summary of CNVs identified in P7 WT and *Top1* cKO neurons.** Genotype, genomic coordinates, cell name, copy number and size of every CNV identified in P7 WT and *Top1* cKO cortical neurons.
