# Supplementary material for: Deletion of Topoisomerase 1 in excitatory neurons causes genomic instability and early onset neurodegeneration
Source: Nat Commun. 2020 Apr 23;11:1962. doi: 10.1038/s41467-020-15794-9 (PMC7181881; doi:10.1038/s41467-020-15794-9)
Supplement: Supplementary file 7 — Reporting Summary [file 41467_2020_15794_MOESM7_ESM.pdf]

natureresearch

Corresponding author(s): Mark Zylka

Last updated by author(s): 2020/03/17

Reporting Summary

Nature Research wishes to improve the reproducibility of the work that we publish. This form provides structure for consistency and transparency in reporting. For further information on Nature Research policies, see Authors & Referees and the Editorial Policy Checklist .

Statistics

For all statistical analyses, confirm that the following items are present in the figure legend, table legend, main text, or Methods section.

n/a	Confirmed	
☐	☒	The exact sample size (*n*) for each experimental group/condition, given as a discrete number and unit of measurement
☐	☒	A statement on whether measurements were taken from distinct samples or whether the same sample was measured repeatedly
☐	☒	The statistical test(s) used AND whether they are one- or two-sided<br>*Only common tests should be described solely by name; describe more complex techniques in the Methods section.*
☒	☐	A description of all covariates tested
☐	☒	A description of any assumptions or corrections, such as tests of normality and adjustment for multiple comparisons
☐	☒	A full description of the statistical parameters including central tendency (e.g. means) or other basic estimates (e.g. regression coefficient) AND variation (e.g. standard deviation) or associated estimates of uncertainty (e.g. confidence intervals)
☐	☒	For null hypothesis testing, the test statistic (e.g. *F*, *t*, *r*) with confidence intervals, effect sizes, degrees of freedom and *P* value noted<br>*Give P values as exact values whenever suitable.*
☒	☐	For Bayesian analysis, information on the choice of priors and Markov chain Monte Carlo settings
☒	☐	For hierarchical and complex designs, identification of the appropriate level for tests and full reporting of outcomes
☒	☐	Estimates of effect sizes (e.g. Cohen's *d*, Pearson's *r*), indicating how they were calculated

Our web collection on statistics for biologists contains articles on many of the points above.

Software and code

Policy information about availability of computer code

Data collection	Western blot fluorescent images were captured using Image Studio Software (Odyssey). Immunostaining and in situ hybridization images were captured using Zeiss ZEN Image Acquisition software. NAD+ quantification were collected using Synergy 2 microplate reader (Biotek) and using Gen5 (version 2.0) software. FACS data were acquired using Summit 5.2. PET images were reconstructed using the 2D-OSEM algorithms.
Data analysis	For th CNV pilot analysis: BWA (version 0.7.12) was used to align Illumina single-end reads to the genome. Samtools (v1.1) was used to generate BAM files. Picard Tools (v1.105) was used to remove duplicate reads. Bedtools (v2.17.0) was used to count aligned reads in genomic bins. R (v3.4.1) was used to normalize binned read counts to copy number estimates, segment bin values (DNAcopy, v1.50.1), and plot CNV profiles (ggplot2, v2.2.1). R was also used to fit Gaussian distributions to dataset-wide segment data to determine CNV thresholds (mixtools, v1.1.0). For the main CNV analysis, reads were aligned to the mouse genome (mm10) using CellRanger-dna cnv (version 1.1.0). Single cell BED files were extracted from CellRanger output using SAMtools (version 1.9), BAMtools (version 2.5.1), and BEDtools (version 2.15.0) and then were uploaded to Ginkgo for CNV analysis using default parameters. Bin level copy number estimates for all single cells were downloaded from Ginkgo (http://qb.cshl.edu/ginkgo) and plotted with filtered CNV results in R (version 3.4.1) using ggplot2 (version 2.2.1) to generate CNV profiles. For scRNAseq analysis FASTQ files were processed using the Dropseq Toolkit (version 1.2) where possible and aligned to the mouse genome (mm10) using STAR. R (version 3.4.1) was used to filter cells based on transcript levels, normalize expression data to obtain the final gene expression matrix, Louvain clustering to define cell clusters, binomial test to define cluster markers. DESeq2 (version 1.38.0) was used to perform differential expression analysis and resluts were plotted using using ggplot2 (version 2.2.1) in R (version 3.4.1).<br>Fiji with ImageJ (1.0) was used for image analysis and quantitation, GraphPad Prism (7.00) was used for statistical analysis of imaging data, NAD+ quantification, motor function assays, wight measurements and survival data. CellProfiler (3.0) was used to quantify single cell in situ hybridization images.

For manuscripts utilizing custom algorithms or software that are central to the research but not yet described in published literature, software must be made available to editors/reviewers. We strongly encourage code deposition in a community repository (e.g. GitHub). See the Nature Research guidelines for submitting code & software for further information.

Data

Policy information about availability of data

All manuscripts must include a data availability statement. This statement should provide the following information, where applicable:

- Accession codes, unique identifiers, or web links for publicly available datasets
- A list of figures that have associated raw data
- A description of any restrictions on data availability

Single-cell genomic data (Fig. 6d, Supplementary Figure 6e and Supplementary Table S3) are available from the NCBI Sequence Read Archive (https://www.ncbi.nlm.nih.gov/sra/PRJNA548496). Single-cell RNA sequencing data (Fig. 5 a-b-c, Supplementary Figure 5 a-b, Supplementary Tables S1 S2) are available from the NCBI Gene Expression Omnibus database (https://www.ncbi.nlm.nih.gov/geo/query/acc.cgi?acc=GSE146672)
All source data are provided in a Source Data file.

Field-specific reporting

Please select the one below that is the best fit for your research. If you are not sure, read the appropriate sections before making your selection.

☒ Life sciences ☐ Behavioural & social sciences ☐ Ecological, evolutionary & environmental sciences

For a reference copy of the document with all sections, see nature.com/documents/nr-reporting-summary-flat.pdf

Life sciences study design

All studies must disclose on these points even when the disclosure is negative.

Sample size	The sample sizes used are listed in the respective Figures and Figure legends. No statistical methods were used to predetermined the sample size. At least 3 mice per condition and 2 sections per mouse were used for every statistical analysis except for the following. CNV analyses were performed on a total of 2 mice per genotype. Initial CNV analysis was performed on 39 WT and 43 Top1 cKO NeuN+ nuclei extracted from one mouse per genotype. Final CNV analysis was performed on 123 WT and 169 cKO neurons from one mouse per genotype (different from the initial analysis). single-cell RNAseq analysis was performed on 2 WT and 2 cKO mice for a total of 1596 cells (683 WT, 913 cKO). Single-cell in situ hybridization was performed on 11 WT and 14 cKO P7 cortical sections coming from 2 mice per genotype.
Data exclusions	For pilot CNV analysis, WGA quality control using Bayesian Information Criteria (BIC > -1.5) removed 13 cells from subsequent analysis. For main CNV analysis, regions containing 6 or more CNVs were considered to be "hotspots" for false positive CNVs, and any CNVs located entirely within the boundaries of a hotspot were excluded. To further safeguard against false positives, CNVs under 5 Mb in length were also excluded. For single-cell RNAseq analysis cells expressing fewer than 300 different genes or whose mitochondrial transcripts exceeded 10% were excluded. Genes expressed in fewer than 3 cells were removed. No other data were excluded from the analyses. All exclusion criteria were pre-established based on previous work.
Replication	All experiments were replicated at least 3 times and all attempts at replication were successful. The number of replicate experiments and sample used is indicated in the figure legend of each figure
Randomization	No sex-related differences were observed in the phenotype, therefore animals of either sex were randomly chosen to perform the experiments
Blinding	All data analysis were performed in blind of the genotype. Data collection was performed in blind of the genotype up to P7, when the genotype of the Top1 cKO animals was visually evident. Samples were labeled with codes to hide genotypes and allow blind data analysis.

Reporting for specific materials, systems and methods

We require information from authors about some types of materials, experimental systems and methods used in many studies. Here, indicate whether each material, system or method listed is relevant to your study. If you are not sure if a list item applies to your research, read the appropriate section before selecting a response.

Materials & experimental systems

n/a	Involved in the study	
☐	☒	Antibodies
☒	☐	Eukaryotic cell lines
☒	☐	Palaeontology
☐	☒	Animals and other organisms
☒	☐	Human research participants
☒	☐	Clinical data

Methods

n/a	Involved in the study	
☒	☐	ChIP-seq
☐	☒	Flow cytometry
☒	☐	MRI-based neuroimaging

Antibodies

Antibodies used

For WB analysis: rabbit anti-PARP (1:1000, Cell Signaling, 9532), rabbit anti-PAR/pADPr (1:1000, R&D Systems, 4336-APC-050), mouse anti-GAPDH (1:1000, Thermo Fisher, MA5-15738), anti-mouse IRdye 680 (1:10000 Licor Odyssey, 925-68070), donkey anti-rabbit IRDye 800CW (1:10000 Licor Odyssey, 926-32213). For immunostaining: rabbit anti-Topoisomerase 1 (1:300; GeneTex, GTX63013, EPR5375), guinea pig anti-NeuN (1:400; EMD-Millipore, ABN90P), rabbit anti-Cux1 (1:200; Santa Cruz Biotechnology, sc-13024), rat anti-Ctip2 (1:200; Abcam, ab18465), rabbit anti-Cleaved-Caspase-3 (1:100; Cell Signaling Technology, 9664), rabbit anti-Iba1 (1:400; Wako, 019-19741), goat anti-GFAP (1:750; Abcam, ab53554), and rabbit anti-phospho-Histone H2A.X (1:50; Cell Signaling Technology, 2577), Phospho-53BP1 (Ser1778) (1:50, Cell Signaling Technology, 2675), anti-rabbit IgG Alexa 488 or Alexa 568 (Thermo Fisher Scientific, A21206 and A10042, respectively), donkey anti-goat IgG-Cy3 (Jackson ImmunoResearch Laboratories, 705-165-003), donkey anti-rat IgG-Cy3 (Jackson ImmunoResearch Laboratories, 712-165-153), and donkey anti-guinea-pig IgG-Alexa 647 (Jackson ImmunoResearch Laboratories, 706-605-148). For FACS: anti-human NeuN IgG clone A60 Alexa Fluor 555 conjugate (1:250, Millipore, MAB377)

Validation

For WB analysis:
rabbit anti-PARP (1:1000, Cell Signaling, 9532) tested for WB/IF/FACS by manufacturer and used in over 250 publications
rabbit anti-PAR/pADPr (1:1000, R&D Systems, 4336-APC-050), tested for WB by manufacturer and used in 7 publications PMID: 27053772
mouse anti-GAPDH (1:1000, Thermo Fisher, MA5-15738), tested for WB/IF/FACS by manufacturer and used in over 250 publications

anti-mouse IRdye 680 (1:10000 Licor Odyssey, 925-68070),
donkey anti-rabbit IRDye 800CW (1:10000 Licor Odyssey, 926-32213).
All secondary antibodies were tested for WB by manufacturer and used in over 250 publications

For immunostaining:
rabbit anti-Topoisomerase 1 (1:300; GeneTex, GTX63013, EPR5375), tested by IF/WB by us using genetic Top1 ko mice, by the manufacturer and used in over 15 publications.
guinea pig anti-NeuN (1:400; EMD-Millipore, ABN90P), tested by us for cell specificity by IF in cortical sections, tested by the manufacturer by WB/IF and used in 4 publications
rabbit anti-Cux1 (1:200; Santa Cruz Biotechnology, sc-13024), tested for IF by us for neuronal specificity in brain sections, tested by the manufacturer by WB/IF and used in 6 publications
rat anti-Ctip2 (1:200; Abcam, ab18465), tested for IF by us for neuronal specificity in brain sections, tested by the manufacturer by WB/IF and used in 362 publications
rabbit anti-Cleaved-Caspase-3 (1:100; Cell Signaling Technology, 9664), tested for WB/IF/FACS by manufacturer and used in 1911 publications
rabbit anti-Iba1 (1:400; Wako, 019-19741), tested for WB/IF by manufacturer and used in 910 publications
goat anti-GFAP (1:750; Abcam, ab53554), tested for WB/IF by manufacturer and used in 118 publications
rabbit anti-phospho-Histone H2A.X (1:50; Cell Signaling Technology, 2577), tested for WB/IF/FACS by manufacturer and used in 438 publications
Phospho-53BP1 (Ser1778) (1:50, Cell Signaling Technology, 2675), tested for WB/IF/FACS by manufacturer and used in 19 publications

anti-rabbit IgG Alexa 488 or Alexa 568 (Thermo Fisher Scientific, A21206 and A10042, respectively), donkey anti-goat IgG-Cy3 (Jackson ImmunoResearch Laboratories, 705-165-003), donkey anti-rat IgG-Cy3 (Jackson ImmunoResearch Laboratories, 712-165-153), and donkey anti-guinea-pig IgG-Alexa 647 (Jackson ImmunoResearch Laboratories, 706-605-148).

All secondary antibodies have been tested by the manufacturers and affinity-purified to minimize cross-reactivity. We routinely perform a negative control with only the secondary antibody to test for specificity. All the secondary antibodies used were specific.

For FACS:
anti-human NeuN IgG clone A60 Alexa Fluor 555 conjugate (1:250, Millipore, MAB377), tested for WB/IF by manufacturer and used in 1084 publications

Animals and other organisms

Policy information about studies involving animals; ARRIVE guidelines recommended for reporting animal research

Laboratory animals

Animal used in this study were mice. Neurod6-Cre mice and p53 -/- mice were obtained from Jackson Laboratory. Top1 fl/fl conditional mice were generated in our lab as described in Mabb et al. 2016. Top1 cHET and cKO were obtained by crossing Neurod6-Cre mice eith Top1 fl/fl mice. Ages range between P0 and P100. Both males and females were used for these studies

Wild animals

The study did not involve wild animals.

Field-collected samples

The study did not involve samples collected from the field.

Ethics oversight	All animal procedures were approved by the University of North Carolina at Chapel Hill Animal Care and Use Committee. Protocol # 18-183

Note that full information on the approval of the study protocol must also be provided in the manuscript.

Flow Cytometry

Plots

Confirm that:

- [x] The axis labels state the marker and fluorochrome used (e.g. CD4-FITC).
- [x] The axis scales are clearly visible. Include numbers along axes only for bottom left plot of group (a 'group' is an analysis of identical markers).
- [x] All plots are contour plots with outliers or pseudocolor plots.
- [x] A numerical value for number of cells or percentage (with statistics) is provided.

Methodology

Sample preparation	P7 cortices were isolated by careful dissection with the hippocampus removed and frozen at -80 C degrees. Nuclei were purified using an iodixonol cushion and neuronal nuclei (NeuN positive/SYTO 13 positive) were isolated using FACS
Instrument	Beckman Coulter MoFlo XDP
Software	Data collection with Summit 5.2
Cell population abundance	NEUN positive neurons were about 10% of total events
Gating strategy	Negative populations for SYTO 13 and NEUN were identified with the use of unstained samples. Nuclei were distinguished from debris by plotting SYTO 13 on the y axis and FSC on the x axis and drawing a gate around SYTO 13 positive events. Singlets were selected by plotting FSC-W vs. FSC-A. SYTO 13 positive nuclei were then plotted for NEUN expression (y axis) and FSC (x axis). NEUN positive and negative populations were clearly distinguishable and showed a 10x difference in the fluorescence intensity of the NEUN channel.

- [x] Tick this box to confirm that a figure exemplifying the gating strategy is provided in the Supplementary Information.
